# Supplementary material for: Similarity in viral and host promoters couples viral reactivation with host cell migration
Source: Nat Commun. 2017 May 2;8:15006. doi: 10.1038/ncomms15006 (PMC5418578; doi:10.1038/ncomms15006)
Supplement: Supplementary Information — Supplementary Figures, Supplementary Tables, Supplementary Note and Supplementary References [file ncomms15006-s1.pdf]

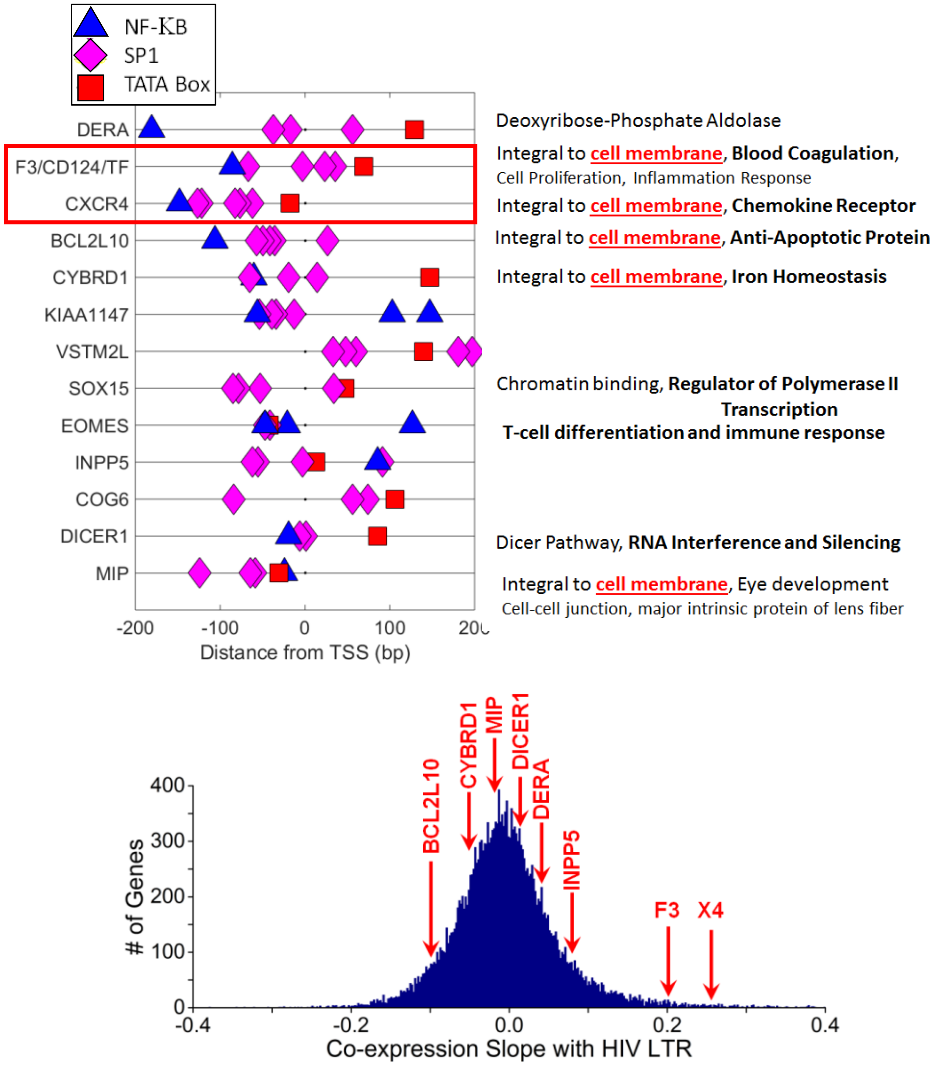

Supplementary Figure 1: Human core promoters with highest *cis* regulatory similarity to the HIV LTR.

(upper) These *cis* binding site arrangements represent the most similar promoters to the HIV LTR found in the genome-wide human promoter comparison after a logic-based search and manual comparison of the promoter group resulting from the search for closest similarity in a range of +/-250bp. The binding sites displayed are the raw results from our search before any alignment between the promoters based on TATA box positioning or multiple TSSs of the genes. Promoters with higher similarity are grouped towards the top. In addition to F3 and CXCR4 studied and discussed in the main text (highlighted with a red box), promoter similarities including TATA-SP1-NFKB arrangments include DERA, and BCL2L10. Not only do many of these promoters serve important functions within the body, such as coagulation, homing, and apoptosis, but are also important functions for viral pathogenesis and fitness. Many of these promoters produce gene products that are integral to the cell membrane, although gene ontology analysis of the full gene list in Supplementary Table 1 does not show specific

enrichment for membrane bound genes. F3 and CXCR4 promoter arrangements are compared to HIV in Figure 1b where the F3 promoter in Figure 1 has been published in an extensive promoter study in the literature[1] and closely matches the above version from our search. **(lower)** Despite having regulatory similarity with regards to the presence of TATA-SP1-NFKB sites (without necessary similarity in arrangement, number of sites, and distances from the TSS), unlike CXCR4 and F3 which have high co-expression slopes, these promoters displayed a range of co-expression slopes when compared with the HIV LTR as in Figure 2. Genes labeled on the histogram are all of the genes from the promoter subgroup that exist in the Lamb et al., 2006 microarray dataset[2]. The range of resulting co-expression slopes supports that existence of the three *cis* element types is insufficient for high co-expression seen with CXCR4 and F3, and further supports their selection as genetically coupled, co-regulated, and among the most co-expressed promoters with the HIV LTR for phenotypic investigation in the study.

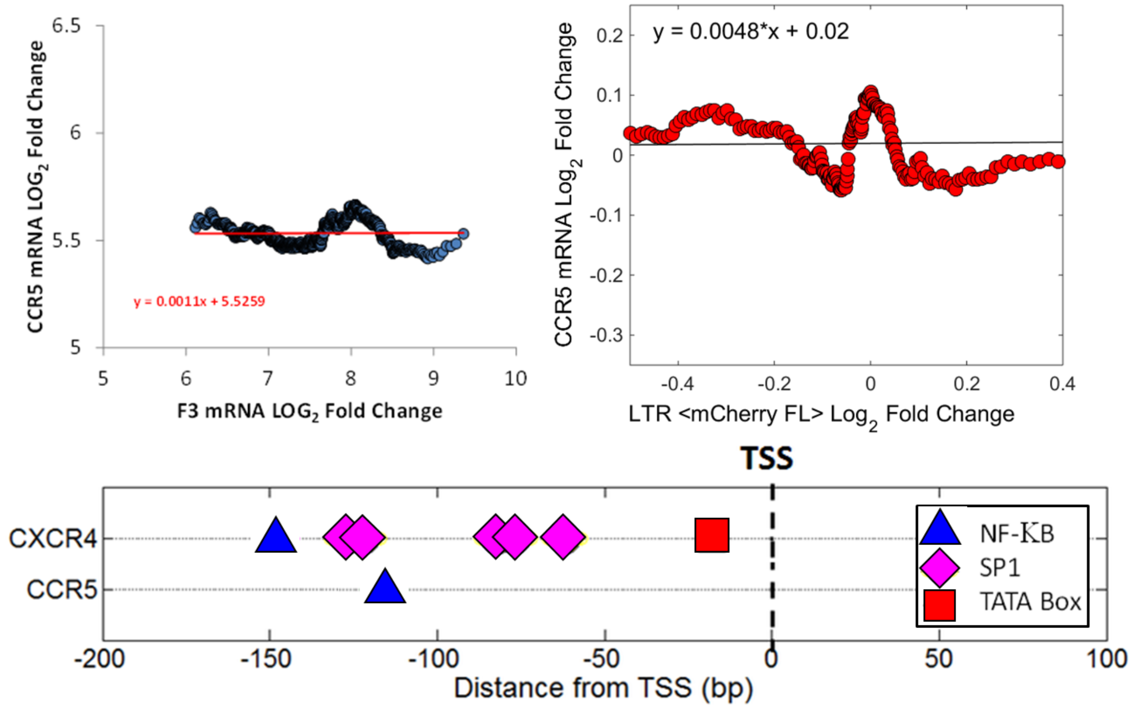

Supplementary Figure 2: Genetically coupled viral-host promoters show no co-expression with human CCR5.
Unlike the strong co-expression observed by both F3 and LTR to CXCR4, a secondary tropism typically seen at later stages of HIV infection[3], both F3 and LTR promoters show no co-expression with CCR5 across hundreds of perturbation microarrays in HL60 cells[2] **(upper left)** and between the HL60 microarrays and the LTR drug screen in Jurkat T-cells[4] **(upper right)**. Comparison of the CCR5 promoter architecture shows that with only a single NFKB binding site it is dissimilar to CXCR4, F3, and the LTR **(lower)**.

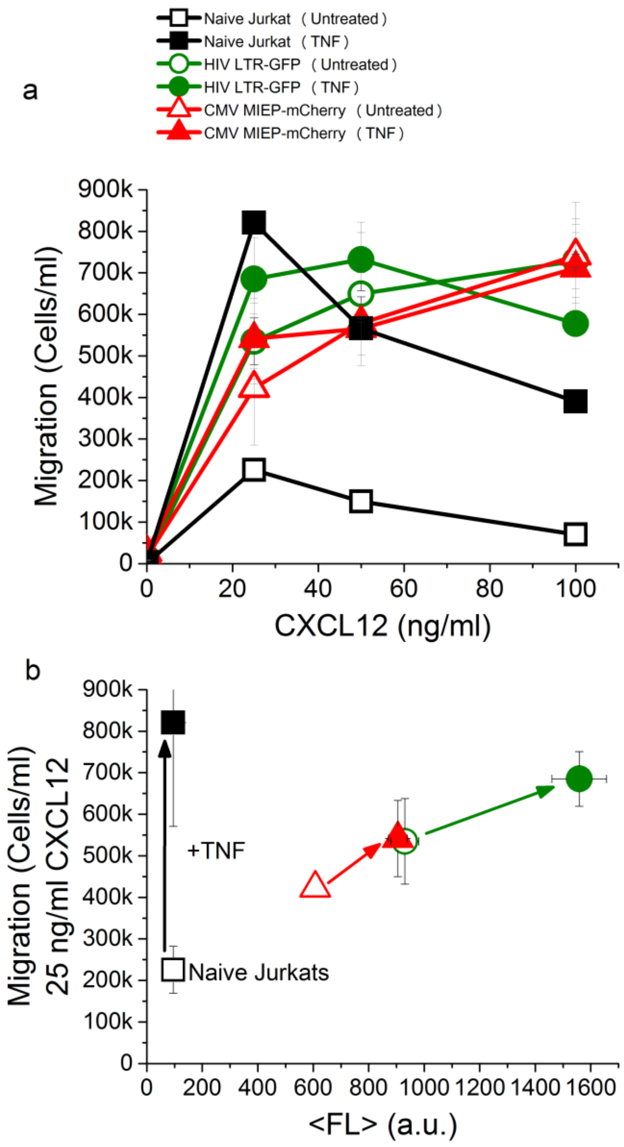

Supplementary Figure 3: Viral gene expression and cell migration increase with TNF treatment.

(a) CXCL12 dose response curves of naïve Jurkat cells, polyclonal HIV-1 LTR driving GFP (LTR-GFP), and the CMV MIEP driving mCherry (MIEP-mCherry)[5] reveal increased migration with CXCL12 concentrations ranging 0-100 ng/mL after 24h TNF treatment. CXCL12 concentration of 25 ng/ml is sufficient for a significant increase of migration for TNF-treated cells (filled symbols) compared to untreated cells (empty symbols). This concentration was used for all migration experiments. **(b)** LTR-GFP and MIEP-mCherry show correlated gene expression and migration after 24h TNF. Naïve Jurkat cells show only an increase in migration with TNF. All measurements of gene expression and migration were performed in duplicate. Migration assays were performed using a 96-well plate and the average values and standard-error are plotted.

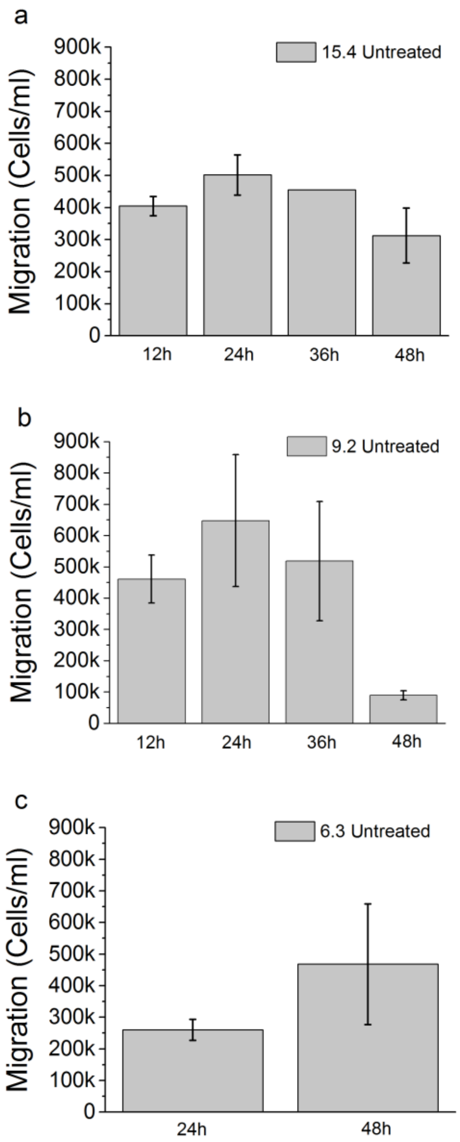

Supplementary Figure 4: Migration of untreated latently infected Jurkat populations. Average migration values for untreated JLat clones **(a)**15.4, **(b)** 9.2, and **(c)** 6.3 after 12-48h of TNF-treatment. JLat 6.3 was not measured for 12h or 36h timepoints. Figure 4b of the main text contains the 12h time point for 15.4 and 9.2 as well as the 24h time point for 6.3. Untreated migration remains fairly constant for 12-36h and deviates in random directions among the clones at 48h. Experiments were performed in a 24-well plate format in duplicate or triplicate and carried out independently on different days. The average value and standard-error for separate duplicates and triplicates are plotted.

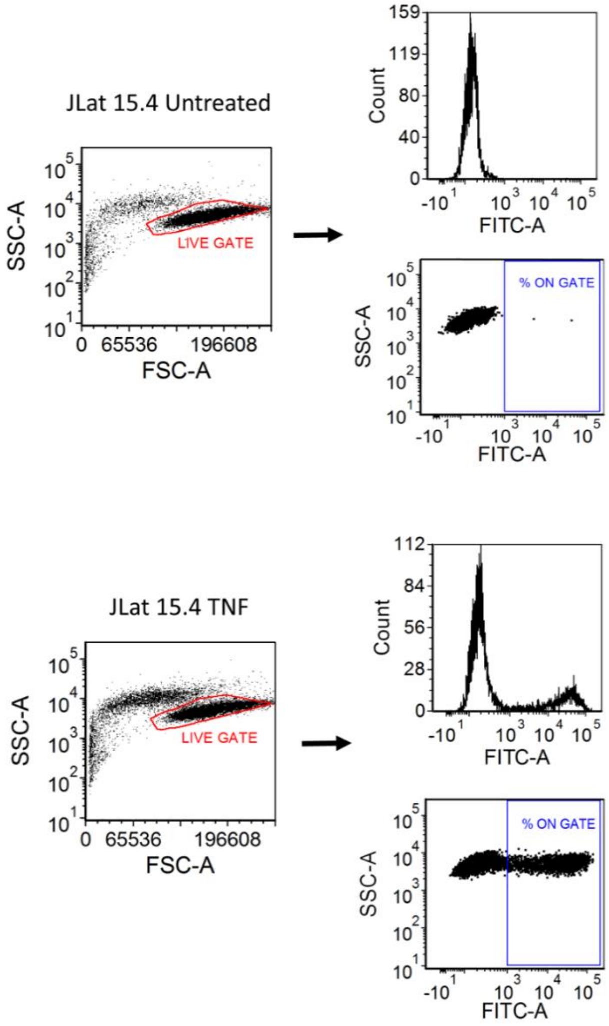

Supplementary Figure 5: Flow cytometry gating strategy for JLat cell measurements. The figure above shows the flow cytometry gating strategy used for the determination of reactivation in Jurkat cells latently-infected with HIV. 10k cells were collected for each measurement from either untreated (upper) or drug treated (lower) JLat isoclones and primary cells. Forward versus side-scatter of cells (left) are displayed along with the conservative gating of LIVE cells (in red). A gate for reactivation in the SSC-A vs. FITC-A scatter plot (blue gate, right panel) is created using the untreated measurement (upper). The “ON GATE” describes the percentage of reactivated cells among the LIVE cell population. A similar ON GATE procedure was used for unsorted CD4+ primary cell experiments, and for ON sorted primary cell experiments, mean fluorescence was determined using the LIVE Gate.

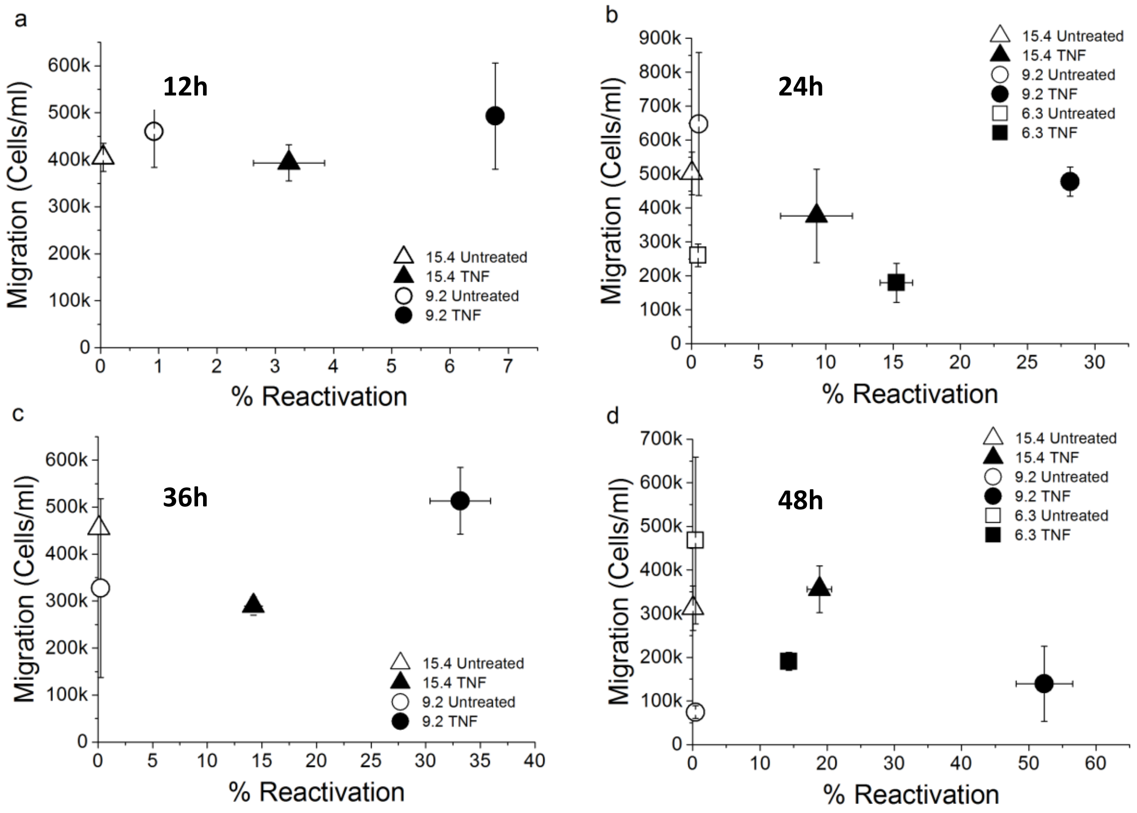

Supplementary Figure 6: Full-length JLat clones decouple CXCR4-LTR co-expression for different durations of TNF treatment.

The plots represent the average values of migration and reactivation for the untreated and TNF-treated JLat isoclones 9.2, 15.4. and 6.3 after **(a)** 12h, **(b)** 24h, **(c)** 36h and **(d)** 48h of treatment. JLat 6.3 was not measured for 12 and 36h treatments. The values for migration of untreated JLats (12h to 48h) can be found in Supplementary Figure 4. The 12h and 24h time points of untreated JLats and all time points for TNF-treated JLats are found in Figure 4b of the main text. For all JLats at all time points, reactivation with TNF shows either a conservation or decrease of migration levels supporting that the full-length virus including Tat expression decouples CXCR4-LTR co-expression seen in Figure 4a and Supplementary Figure 3. JLat isoclone 15.4 shows reduced or similar migration rates after 12h, 24h and 36h of treatment compared to the untreated population. JLat 6.3 reveals reduced migration rates after all TNF-treatments. Similar migration rates can be observed for JLat 9.2 after 12h, 36h and 48h of treatment as well as for JLat 15.4 after 48h of treatment with TNF. Migration assays were performed in triplicate using a 24-well migration plate and the average values are plotted with standard-error.

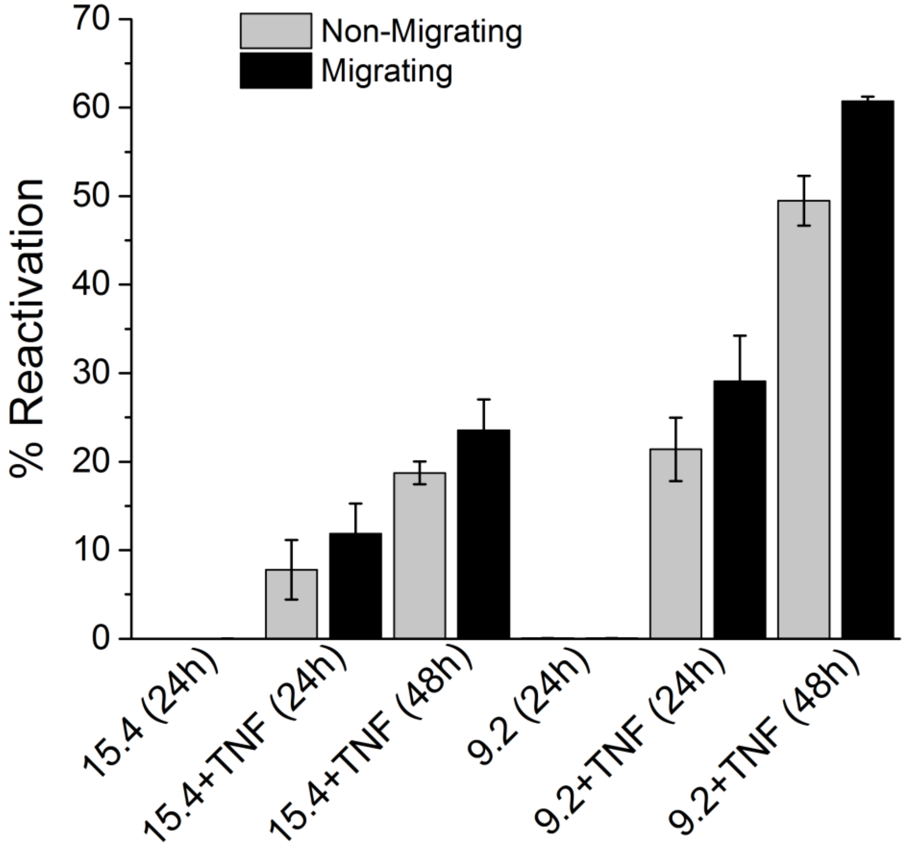

Supplementary Figure 7: Reactivation of migrating and non-migrating cells of JLat isoclone 9.2 and 15.4 after 24h and 48h of TNF-treatment.

To test for any time dependence in the effect seen in Figure 4e, with migrating cells reactivating more than non-migrating cells, measurements after 24h treatment were performed for comparison to 48h. Consistent increases in reactivation were observed for both JLat isoclones despite having overlapping standard-error bars. The increase of reactivation is further accentuated for 48h TNF. The results reveal that migrating cells consistently reactivate more compared to non-migrating cells with higher reactivation rate as the percentage of “ON” cells is consistently higher over time (see section on reactivation rate calculation above). Migration assays were performed in duplicate and triplicate for 24h and 48h respectively using a 24-well migration plate, and average values are plotted with standard-error.

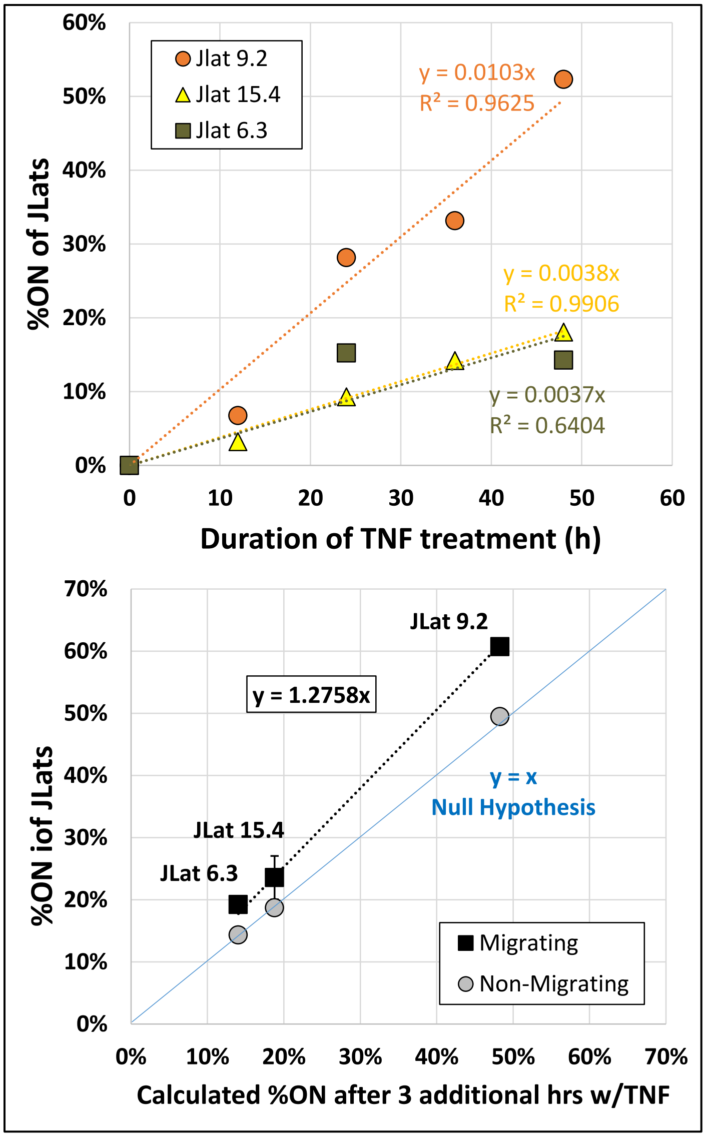

Supplementary Figure 8: JLat reactivation rates and calculated reactivation for migrating cells. (upper) Calculation of reactivation rates for each of three JLat clones based on an observed linear trend for reactivation over time. **(lower)** Comparison of reactivated cells versus their expected amount of reactivation using a constant reactivation rate for migrating and non-migrating populations (*Null hypothesis*, blue line). Migrating cells land above the line with increased reactivation than expected while the non-migrating population represents the constant rate assumption during the migration experiment. For additional calculation details see Supplementary Note 1.

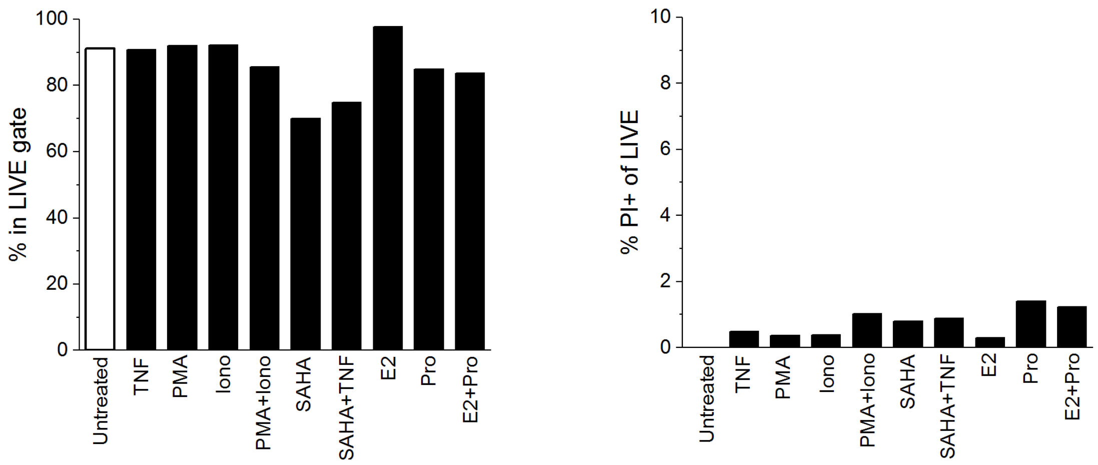

Supplementary Figure 9: Propidium iodide staining of treated activated and uninfected primary CD4+ T-cell populations shows minimal drug toxicity post-48h.
Cell death staining post-48h drug treatments on activated and uninfected primary CD4+ T-cells was performed to confirm that cell death was minimal for treatments in Figures 4-6. Propodium iodide (PI) staining, using drug concentrations listed in Supplementary Table 2, shows that within the live gated cell population of the flow cytometer **(left)**, all cells remain below a PI+ value of 2% **(right)** indicating that a large majority of cells in the live gate are indeed live. This is consistent with the drug concentrations selected based on previous studies[6-14] as well as previous PI staining of treated cells[4].

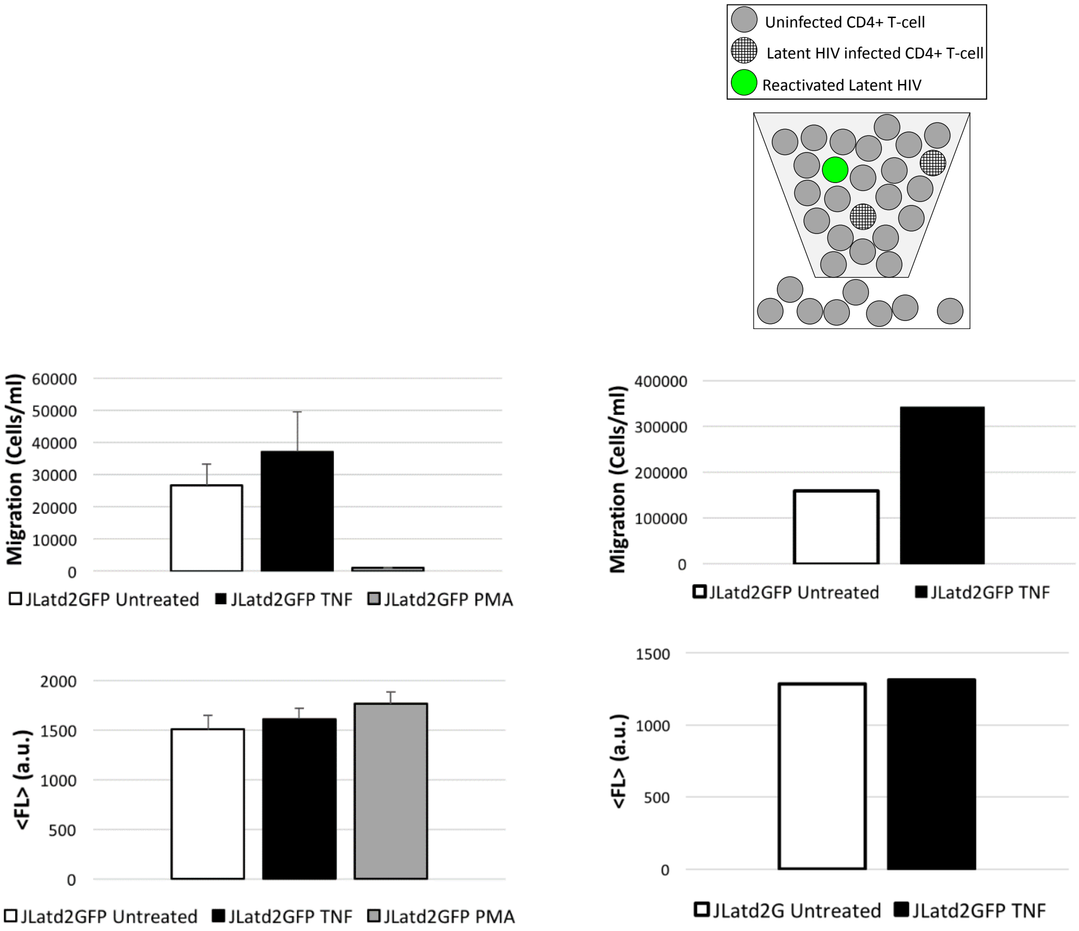

Supplementary Figure 10: Unsorted and OFF sorted HIV infection of activated primary CD4+ T-cells is insufficient to detect decoupling of migration.
Activated primary CD4+ T-cells were infected with the JLatd2GFP construct using spinoculation. For the unsorted cell population (left panels), the decoupling of increased migration post-48h TNF treatment is no longer discernible due to the dominating number of uninfected CD4+ T-cells which obscure the the composite behavior with the HIV infected cells **(upper left)**. Mean expression of the total population remains fairly constant with uninfected cells dominating fluorescence levels **(lower left)**. PMA treatment shows quenching of CXCR4-mediated migration consistent with Figures 5 and 6, suggesting off-target migratory suppression by PMA seen in Supplementary Figure 8. Similarly, OFF, GFP- sorted CD4+ T-cells infected with JLatd2GFP show similar behavior with a strong increase in migration with 48h TNF treatment, and any potential latent minority of cells are unable to decouple the migration increase caused by drug treatment **(upper and middle right)**. Mean fluorescence of the OFF, GFP- sorted cell population is uniform and no difference is viewable among untreated and TNF-treated cell populations **(lower right)**. These results present a challenge in running migration experiments for non-pure latent OFF-sorted primary cell populations, typically consisting of a very small minority of latent cells selected by sorting the GFP- infected population and which is dominated by uninfected cell behavior **(upper right)**. All experiments with unsorted primary CD4+ T-cells were performed as a single measurement using 2 donors and the average values are plotted with standard-error. However, experiments with OFF-sorted primary CD4+ T-cells were carried out as a single measurement using a single donor. Infection of CD4+ T-cells was quantified at

~35% GFP+ cells whereby the rate of reactivation was similar between untreated and TNF-treated cells.

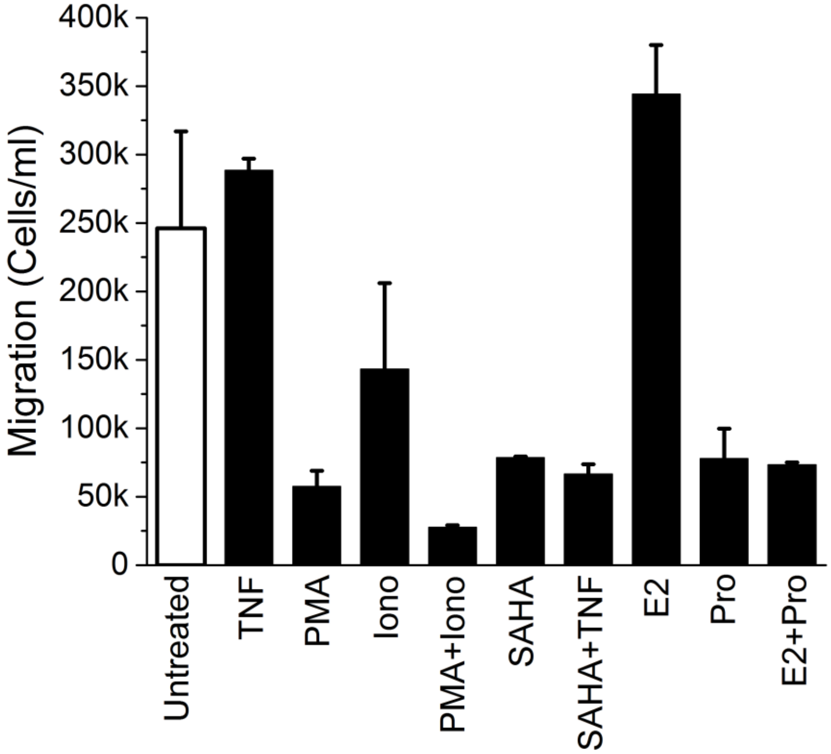

Supplementary Figure 11: Effect of drug treatments on migration of uninfected primary CD4+ T-cells.
Isolated, activated, and uninfected CD4+ T-cells were treated for 48h with diverse drugs pre-migration. 17ß-Estradiol (E2) shows increases in migration compared to the untreated population, PMA reduces migration alone and with Ionomycin addition. Similarly, SAHA combined with TNF reduces TNF migration, and Prostratin (Pro) reduces enhanced E2 migration. All measurements were performed in duplicate on a single donor and the average values are plotted with standard-error.

Supplementary Table 1: List of human promoters that pass search similarity to HIV LTR promoter with TBP-SP1-NFKB binding sites.

(366 Total Gene Symbols)

Symbol	Symbol	Symbol	Symbol	Symbol	Symbol	Symbol	Symbol
ACBD5	C20orf29	DHH	HYI	MIR1281	PPP1R15A	SOX15	ZNF550
ADORA1	C3orf52	DICER1	ICA1	MIR3175	PRDX3	SPSB3	ZNF611
AF279782	C6orf165	DKFZp451B082	IDAS	MIR4472-2	PRKCDBP	SQRDL	ZNF642
AFAP1L2	C9orf72	DL492006	IDS	MIR484	PRRG4	SSR3	ZNF662
AGMAT	CA13	DMRT2	IFT43	MMAB	PSD	ST8SIA4	ZNF669
AK023629	CACNB2	DNAH3	IFT57	MOGS	QDPR	STK32C	ZNF670
AK054845	CBLN4	DNAJC8	IL23A	MRPS5	R3HDM2	STRADB	ZNF678
AK093279	CCDC107	DNPEP	INPP5F	MT1G	RBM4B	SVEP1	ZNF695
AK094859	CCDC11	DRG1	KAZALD1	MT3	RDM1	SYF2	ZNF695
AK297461	CCDC120	DTNBP1	KEL	MTIF3	RGS16	SYS1	ZNF737
AK5	CCDC67	DYRK3	KIAA0196	MTPAP	RGS2	TCF4	ZNF765
AKAP7	CCDC79	EGLN3	KIAA1002	MTRF1L	RHOA	TDP2	ZNF765
AKNAD1	CCNF	EIF1AY	KIAA1147	MUC6	RICTOR	TFPI2	ZNF791
ALDH1L1	CCNG2	ELP3	KLK3	MYBPH	RIIAD1	TGFB2	ZNF800
ALS2	CCNT2	EMILIN1	LACE1	MYH11	RIPPLY2	TIMELESS	ZNF845
ANAPC16	CD47	ENO1-AS1	LAMB2	MYSM1	RND3	TIMM44	ZNF860
ANGPTL6	CD59	ENTPD4	LAMB2	NBL1	RNF114	TIPRL	
ANKRD52	CD72	EOMES	LAMP5	NDRG3	RPN2	TIRAP	
ANXA7	CDC25B	EP300	LCP1	NEFH	RSL1D1	TM2D3	
ARHGEF12	CDC7	EPC1	LCTL	NELL2	RUNX3	TMEM223	
ARL5B	CDH10	EPHX2	LINC00303	NFKBIL1	S100A5	TMEM237	
ARRDC2	CELF3	EYA1	LLGL2	NGLY1	SAMD5	TMEM55B	
ASPSCR1	CENPO	F3	LOC100125556	NHLRC2	SCGN	TMEM86B	
ATF6B	CHI3L1	FAM105B	LOC100132354	NIPSNAP3B	SCN1B	TMLHE	
ATG13	CKAP2L	FAM136A	LOC100289495	NKX2-6	SCRN1	TNFRSF11A	
ATOH8	CLCC1	FAM49B	LOC100505619	NPAS4	SCYL2	TNFSF15	
ATP1A4	CNNM4	FAM86C1	LOC100505817	NRBP2	SDC4	TRIM14	
ATP5E	CNOT2	FBXL15	LOC100506068	NTF3	SDK1	TRIM24	
ATRIP	COG6	FERMT3	LOC100506779	NUDT12	SEMA4A	TRIM33	
B3GAT3	COPS4	FLT1	LOC100507217	NXT2	SEMA6D	TTYH1	
B3GNT1	CPXM1	FUT1	LOC100507217	ODF3B	SENP7	TUBA1C	
B4GALT5	CTNS	GALNTL6	LOC151174	OIP5	SETBP1	UBB	
BC038465	CTTNBP2	GCET2	LOC284385	PAICS	SFMBT2	UBC	
BC039477	CUL2	GHITM	LOC286467	PAX2	SFRP4	UEVLD	
BC039521	CUX1	GLYR1	LOC400891	PCP2	SGOL1	UNC80	
BC040219	CXCL6	GMDS	LOC400958	PDHB	SIRT5	URB2	
BC142949	CXCR4	GMFG	LOC643387	PEX19	SLC16A3	USP16	
BCL2L10	CYBRD1	GNPDA1	LOC84856	PGBD3	SLC20A2	VCL	
BEX2	CYP51A1	GOT2	LRRC48	PGM2	SLC25A29	VPS18	
BHLHB9	D28390	GSR	MANEA	PHIP	SLC27A5	VSTM2A	
BMP4	DAAM2	GSX2	MAP3K13	PICK1	SLC29A2	VSTM2L	
BOD1	DAXX	GTPBP10	MCFD2	PIGV	SLC2A6	WBSCR27	
BRIP1	DCAF8	HAND2	MCM10	PLAGL1	SLC35D2	WDR44	
BSCL2	DCBLD2	HARBI1	MCM9	PLDN	SLC7A3	WDR52	
C11orf65	DCTPP1	HECTD1	MECOM	PLEKHM2	SLITRK1	ZBTB45	
C12orf29	DDR1	HEY2	MEST	PLK4	SLMO2	ZNF140	
C16orf46	DDX58	HLCS	MFSD2A	PNMA1	SLMO2-ATP5E	ZNF195	
C1D	DERA	HNRNPC	MGC12916	POC5	SMC3	ZNF259	
C1orf123	DGKD	HP1BP3	MICAL2	PODNL1	SNX15	ZNF264	
C1orf52	DGUOK	HTR1A	MIP	POLDIP3	SNX4	ZNF490	

Supplementary Table 2: Summary of drugs used for migration-reactivation cocktail assay on latent HIV (JLat 15.4) in Figure 5.

The following table includes the drug cocktails used, final concentrations, known bioactivities concerning migration and reactivation, measured values for migration and reactivation after 48h treatment of JLat 15.4 (Figure 5), and the % of LIVE cells after 48h drug treatment using flow cytometry live gating. (* - for PI staining see Supplementary Fig. 7). All refrences support the bioactivities listed.

Drug cocktail	Drug	Final concentration	Bioactivities concerning migration and reactivation	Migration level after 48h (in cells/ml)	Reactivation level after 48h	Percentage of cells in the LIVE gate*
Untreated	-	-	-	351167	0.03%	71.1%
Migration	Cytarabine	0.35 µM	Cytosine arabinoside incorporated into human DNA and kills cells	251500	0.1%	30.48%
	17ß-Estradiol **(Kubarek et al. 2007)**	10 µM	Demethylation of CXCR4 promoter	638000	0.04%	55.64%
	17ß-Estradiol + Tamoxifen	10 µM each	Change the cellular milieu that maintains the hypermethylated stage of CpG islands of CXCR4 and CXCL12 promoters	103800	0.07%	40.11%
	Ionomycin **(Huang et al. 2014)**	1 µM	Increase of intracellular calcium level and activation of NFAT (Nuclear factor activated in T-cells)	922500	0.03%	83.45%
	Tamoxifen **(Kubarek et al. 2009)**	10 µM	Antagonist of E2, down-regulation of CXCR4 expression	300500	0.03%	40.99%
	Valproic Acid	1 mM	Histone deacetylase inhibitor (HDACi)	182000	0.09%	52.54%
	Valproic Acid + Tamoxifen	1 mM + 10 µM	-	256417	0.1%	45.71%
Migration and reactivation	5-Aza-2-deoxycytidine	5 µM	-	92867	1.63%	22.29%
	Prostratin **(Hezareh et al. 2004)**	3 µM	Activates PKC and NFKB	48550	2.11%	74.56%
	5-Aza-2-deoxycytidine + Prostatin	5 µM + 3 µM	DNA methyltransferase inhibitor that can reactivate latent HIV	104250	17.24%	45.84%
	Cytarabine + Prostratin	0.35 µM + 3 µM	-	87950	2.75%	59.72%
	17ß-Estradiol + Prostratin	10 µM + 3 µM	-	102500	2.04%	74.84%
	JQ1	1 µM	Bromodomain inhibitor that reactives HIV transcription	388533	0.15%	54.55%
	JQ1 +TNF	1 µM + 10 ng/ml	-	50350	29.68%	21.46%
	Panobinostat **(Mandawat et al. 2010)**	15nM	Depletion of mRNA and protein level of CXCR4, HDACi	121450	0.27%	35.02%
	Prostratin + TNF	3 µM + 10 ng/ml	-	46150	29.37%	69.75%
	Romidepsin **(Ierano et al. 2013)**	5nM	Increase of CXCL 12-mediated extracellular signal-related kinase (ERK) activation but reduced migration and inhibitor of histone deacetylase	188100	0.19%%	49.32%%
	Suberoylanilide Hydroxamic Acid (SAHA) **(Crazzolara et al. 2002)**	2.5 µM	HDACi	17300	0.62%	30.22%
	Tamoxifen + Prostratin	10 µM + 3 µM	-	66500	1.82%	74.56%
	Tumor necrosis factor alpha (TNF) **(Han et al. 2001)**	10 ng/ml	Activates NFKB	397108	17.9%	60.71%
	Tamoxifen + TNF	10 µM + 10 ng/ml	-	394833	19.76%	54.68%
	17ß-Estradiol + TNF	10 µM + 10 ng/ml	-	349633	17.27%	47.76%
Reactivation	Phorbol 12-myristate 13-acetate (PMA) **(Clift et al. 2014)**	200 ng/ml	Activates PKC	<3000	1.88%	82.21%
	PMA + Ionomycin	200 ng/ml + 1 µM	-	<3000	7.75%	72.09%
	PMA +TNF	200 ng/ml + 10 ng/ml	-	<3000	33.28%	74.96%
	SAHA + TNF	2.5 µM + 10 ng/ml	-	5830	31.97%	33.04%
	Valproic Acid + TNF	1 mM + 10 ng/ml	-	9605	25.93%	40.81%

<u>References within the table</u>: 17ß-Estradiol (E2)[11], Tamoxifen (Tam)[12], Ionomycin (Iono)[10], Prostratin (Pro)[9], Tumor necrosis factor alpha (TNF)[8], Phorbol 12-myristate 13-acetate (PMA)[6], Suberoylanilide Hydroxamic Acid (SAHA)[7], Romidepsin (Romi)[15], and Panobinostat (Pano)[13]. All abbreviations were used in Figure 5 and Supplementary Figures 7 and 8.

Supplementary Note 1:

Reactivation rate from latency of migrating cells is higher than non-migrating cells

Consistent results in Figure 4e suggest that reactivation is higher for migrating than non-migrating cells for latent full-length JLat and minimal LTIG constructs.

To approximate if this is true, the following calculation was performed to disprove the *null hypothesis* that reactivation rates are equal between migrating and non-migrating cells.

1. Using average reactivation values for three JLat clones in Figure 4b, reactivation is linear over 12-48h TNF treatment and the rate was calculated using a linear fit **(upper panel, Supplementary Fig. 8)**. Here reactivation was measured with flow cytometry before the migration experiment.

2. (lower panel, Supplementary Fig. 8) Measured reactivation or %ON of JLats post-48h TNF treatment was used for both migrating and non-migrating cells and compared to an expected or calculated %ON taking into account the total number of migrated cells in each experiment, the reactivation level at the start of migration, and the constant reactivation rates calculated in the previous step for the cells that migrate over a 3h migration experiment.

Formulas used for calculation:

Calculated %ON post-3h migration = %ON at start of migration + % of cells that turn on in the migrated population after 3h

Where because the measurement of reactivation for both migrating and non-migrating cells were performed after migration of cells treated with TNF for 48h, the following is used to back-calculate the initial reactivation levels for both migrating and non-migrating cells at the beginning of the migration experiment.

%ON at start of migration = (%ON for Non-migrating population treated for 48h TNF measured at the end of the 3h of migration – % of cells that turned on in the non-migrating population during the 3h experiment)

Example of expected rate calculation for JLat 9.2 :
% ON of migrating population measured in Figure 4e: 60.71%
% ON of non-migrating population measured in Figure 4e: 49.48%
Reactivation rate increase per hour (upper panel above based on Figure 4b): 1.03%
Migration rate/hr based off of total # of cells migrated post-48hrs: 27.9k/hr
Newly reactivating cells per hour within migrating cell population: 27.9k*1.03%/hr = 287.38 cells/hr

Calculated or expected %ON post-3h migration = (49.48% - (3*1.03%*(300k cells – 3*27.9k cells)/300k cells)) + (3*287.38)/(3*27.9k) = 48.28%

3. The results show that the measured reactivation of migrating cells is higher than the predicted calculation using a constant reactivation rate **(**y=x, **lower panel, Supplementary Fig. 8)**, therefore negating the *null hypothesis*. The non-migrating reactivation matches the calculated value, landing on y=x, and represents the constant reactivation rate assumption.

Supplementary References

1. Oeth, P., Parry, G.C. & Mackman, N. Regulation of the tissue factor gene in human monocytic cells. Role of AP-1, NF-kappa B/Rel, and Sp1 proteins in uninduced and lipopolysaccharide-induced expression. *Arteriosclerosis, thrombosis, and vascular biology* **17**, 365-74 (1997).
2. Lamb, J. *et al.* The Connectivity Map: using gene-expression signatures to connect small molecules, genes, and disease. *Science* **313**, 1929-35 (2006).
3. Bleul, C.C., Wu, L., Hoxie, J.A., Springer, T.A. & Mackay, C.R. The HIV coreceptors CXCR4 and CCR5 are differentially expressed and regulated on human T lymphocytes. *Proc Natl Acad Sci U S A* **94**, 1925-30 (1997).
4. Dar, R.D., Hosmane, N.N., Arkin, M.R., Siliciano, R.F. & Weinberger, L.S. Screening for noise in gene expression identifies drug synergies. *Science* (2014).
5. Teng, M.W. *et al.* An endogenous accelerator for viral gene expression confers a fitness advantage. *Cell* **151**, 1569-80 (2012).
6. Clift, I.C., Bamidele, A.O., Rodriguez-Ramirez, C., Kremer, K.N. & Hedin, K.E. beta-Arrestin1 and distinct CXCR4 structures are required for stromal derived factor-1 to downregulate CXCR4 cell-surface levels in neuroblastoma. *Mol Pharmacol* **85**, 542-52 (2014).
7. Crazzolara, R. *et al.* Histone deacetylase inhibitors potently repress CXCR4 chemokine receptor expression and function in acute lymphoblastic leukaemia. *Br J Haematol* **119**, 965-9 (2002).
8. Han, Y., He, T., Huang, D.R., Pardo, C.A. & Ransohoff, R.M. TNF-alpha mediates SDF-1 alpha-induced NF-kappa B activation and cytotoxic effects in primary astrocytes. *J Clin Invest* **108**, 425-35 (2001).
9. Hezareh, M. *et al.* Mechanisms of HIV receptor and co-receptor down-regulation by prostratin: role of conventional and novel PKC isoforms. *Antivir Chem Chemother* **15**, 207-22 (2004).
10. Huang, K., Kiefer, C. & Kamal, A. Novel role for NFAT3 in ERK-mediated regulation of CXCR4. *PLoS One* **9**, e115249 (2014).
11. Kubarek, L. & Jagodzinski, P.P. Epigenetic up-regulation of CXCR4 and CXCL12 expression by 17 beta-estradiol and tamoxifen is associated with formation of DNA methyltransferase 3B4 splice variant in Ishikawa endometrial adenocarcinoma cells. *FEBS Lett* **581**, 1441-8 (2007).
12. Kubarek, L., Kozlowska, A., Przybylski, M., Lianeri, M. & Jagodzinski, P.P. Down-regulation of CXCR4 expression by tamoxifen is associated with DNA methyltransferase 3B up-regulation in MCF-7 breast cancer cells. *Biomed Pharmacother* **63**, 586-91 (2009).
13. Mandawat, A. *et al.* Pan-histone deacetylase inhibitor panobinostat depletes CXCR4 levels and signaling and exerts synergistic antimyeloid activity in combination with CXCR4 antagonists. *Blood* **116**, 5306-15 (2010).
14. Spina, C.A. *et al.* An in-depth comparison of latent HIV-1 reactivation in multiple cell model systems and resting CD4+ T cells from aviremic patients. *PLoS pathogens* **9**, e1003834 (2013).
15. Ierano, C. *et al.* Histone deacetylase inhibitors induce CXCR4 mRNA but antagonize CXCR4 migration. *Cancer Biol Ther* **14**, 175-83 (2013).
